# Biomechanical analysis of acromioclavicular joint dislocation repair using coracoclavicular suspension devices in two different configurations

**DOI:** 10.1007/s10195-015-0346-y

**Published:** 2015-03-05

**Authors:** Ferran Abat, Juan Sarasquete, Luis Gerardo Natera, Ángel Calvo, Manuel Pérez-España, Néstor Zurita, Jesús Ferrer, Juan Carlos del Real, Eva Paz-Jimenez, Francisco Forriol

**Affiliations:** 1 Department of Sports Orthopaedics, ReSport Clinic, Barcelona, Spain; 2 Department of Traumatology and Orthopaedic Surgery, Hospital de la Santa Creu i Sant Pau, Universitat Autónoma de Barcelona, Sant Quintí, 89, Barcelona, 08026 Spain; 3 Hospital IMED Elche, Alicante, Spain; 4 Shoulder and Elbow Pathology Unit Shoulder of Madrid, Madrid, Spain; 5 Engineering School (ICAI), University of Comillas, Madrid, Spain; 6 Universidad San Pablo CEU, Madrid, Spain

**Keywords:** Acromioclavicular dislocation, Joint, Anatomic repair, Biomechanics

## Abstract

**Background:**

The best treatment option for some acromioclavicular (AC) joint dislocations is controversial. For this reason, the aim of this study was to evaluate the vertical biomechanical behavior of two techniques for the anatomic repair of coracoclavicular (CC) ligaments after an AC injury.

**Materials and methods:**

Eighteen human cadaveric shoulders in which repair using a coracoclavicular suspension device was initiated after injury to the acromioclavicular joint were included in the study. Three groups were formed; group I (*n* = 6): control; group II (*n* = 6): repair with a double tunnel in the clavicle and in the coracoid (with two CC suspension devices); group III (*n* = 6): repair in a “V” configuration with two tunnels in the clavicle and one in the coracoid (with one CC suspension device). The biomechanical study was performed with a universal testing machine (Electro Puls 3000, Instron, Boulder, MA, USA), with the clamping jaws set in a vertical position. The force required for acromioclavicular reconstruction system failure was analyzed for each cadaveric piece.

**Results:**

Group I reached a maximum force to failure of 635.59 N (mean 444.0 N). The corresponding force was 939.37 N (mean 495.6 N) for group II and 533.11 N (mean 343.9 N) for group III. A comparison of the three groups did not find any significant difference despite the loss of resistance presented by group III.

**Conclusion:**

Anatomic repair of coracoclavicular ligaments with a double system (double tunnel in the clavicle and in the coracoid) permits vertical translation that is more like that of the acromioclavicular joint. Acromioclavicular repair in a “V” configuration does not seem to be biomechanically sufficient.

## Introduction

Acromioclavicular (AC) dislocations usually present as the result of a fall that produces trauma to the lateral aspect of the shoulder. It brings about a variable separation of the acromioclavicular joint depending on the degree of damage to the capsule, the acromioclavicular ligaments, as well as the coracoclavicular (CC) ligaments. Rockwood classified them into grades I–VI depending on the severity of the injury and the degree of displacement [1]. Grade I–II injuries are treated conservatively, without surgery, leading to satisfactory results and a return to sporting activity in most cases [2]. The treatment of grade III injuries is controversial. However, surgical treatment is recommended for high-grade lesions IV–VI [3]. Despite their clinical impact, there is still no consensus for the surgical treatment of Rockwood high-grade lesions [4, 5].

From a biomechanical point of view, the importance of the acromioclavicular and coracoclavicular ligaments for maintaining the vertical and horizontal stability of the acromioclavicular joint has been shown [6]. There are many techniques that can be applied to the repair of the AC and CC ligaments in the literature [7, 8]. It is currently popular to perform these repairs in an anatomic way [5, 9].

To replace CC ligaments, some authors advocate using tendons (autograft or allograft) [10], while others perform repairs with synthetic devices [11, 12] which allow for the reduction of the AC joint, with the expectation that these devices might act as scaffolding while the injured ligaments heal.

Synthetic CC suspension devices placed arthroscopically permit the reduction of AC dislocations during the biological healing of the CC ligaments. Among the options for repairs with synthetic devices is anatomic reconstruction with a double tunnel in the clavicle as well as in the coracoid [5]. This technique allows the conoid and trapezoid ligaments to be emulated, and has shown biomechanical advantages [12], but there is also an increased risk of fracture of the clavicle during the construction of two tunnels and an increase in technical difficulty [5]. On the other hand, the isometric approach seeks to restore the anatomy of the conoid and trapezoid ligaments by using a single anchoring stitch in the coracoid at the midway point of the insertion of both ligaments.

The aim of the study reported in this paper was to evaluate the vertical biomechanical behavior of two techniques for the anatomic repair of coracoclavicular ligaments that can be used for the surgical treatment of acromioclavicular dislocations using synthetic CC suspension devices. The hypothesis was that anatomic CC repair with a double tunnel in both the coracoid and clavicle is the repair that comes closest to restoring the natural stability of the AC joint.

## Materials and methods

Eighteen human cadaveric shoulders (9 men, 9 women) from individuals aged 41–63 years (mean 58) were used. All specimens studied were free of systemic diseases or previous acromioclavicular injury. The pieces were stored at −20 °C and subsequently prepared prior to study. Shoulders were sectioned and soft tissue was removed, leaving the bone and ligament structure. The scapula bound to the clavicle with the intact coracoclavicular ligaments and acromioclavicular joint were obtained. In all cases, the ZipTight-type synthetic coracoclavicular suspension device was used (Biomet, Warsaw, IN, USA).

Three groups were formed: group I (*n* = 6), the control group; group II (*n* = 6), repair with a double tunnel in both the clavicle and coracoid (with two CC suspension devices); group III (*n* = 6), repair in a “V” configuration with two tunnels in the clavicle and one in the coracoid (with one CC suspension device).

### Reconstruction techniques

**Fig. 1:**
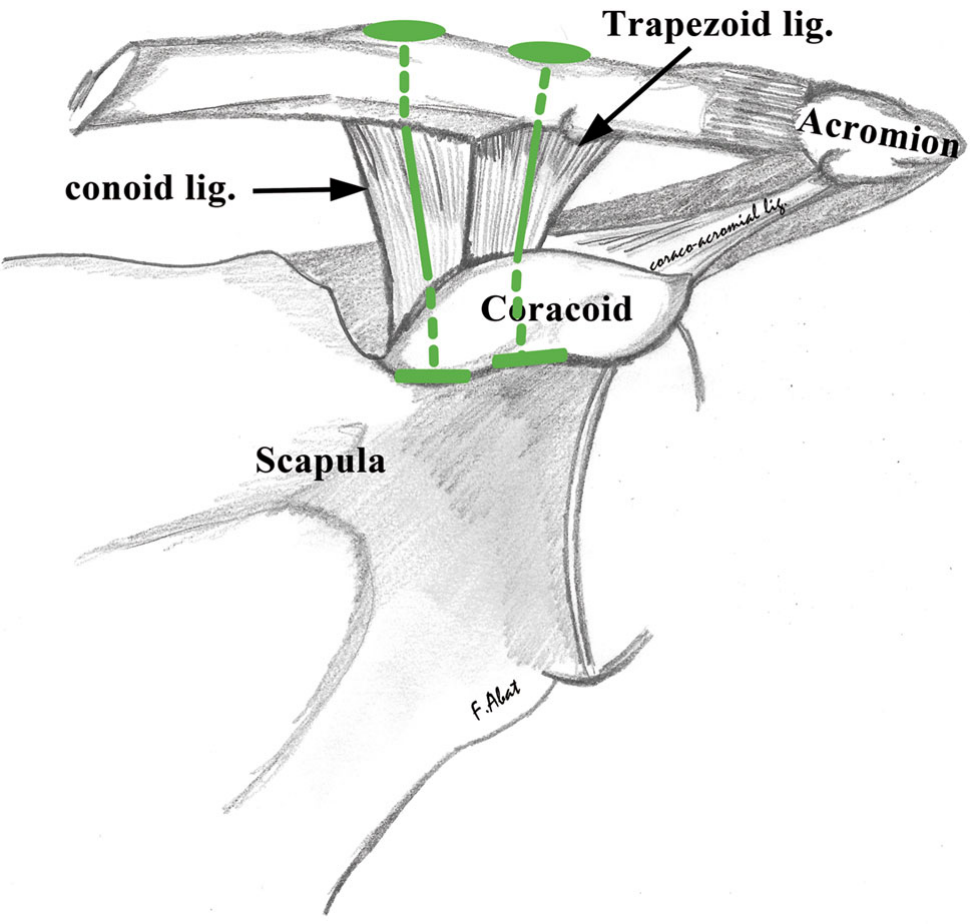
Scheme for anatomic repair of the conoid and trapezoid ligaments with two CC suspension devices. Layout with a double tunnel in both the clavicle and coracoid

For reconstruction with double tunnels in the coracoid (group II), anatomic repair of the CC, conoid, and trapezoid ligaments was performed (Fig. 1). This was done with two tunnels in the clavicle and another two tunnels at the base of the coracoid at the anatomic positions of the ligaments (4.5 cm from the acromial end of the clavicle for the conoid ligament tunnel and 2.5 cm for the trapezoid) [1, 5, 9, 12]. An individualized anatomic ligament repair of each ligament was performed.

**Fig. 2:**
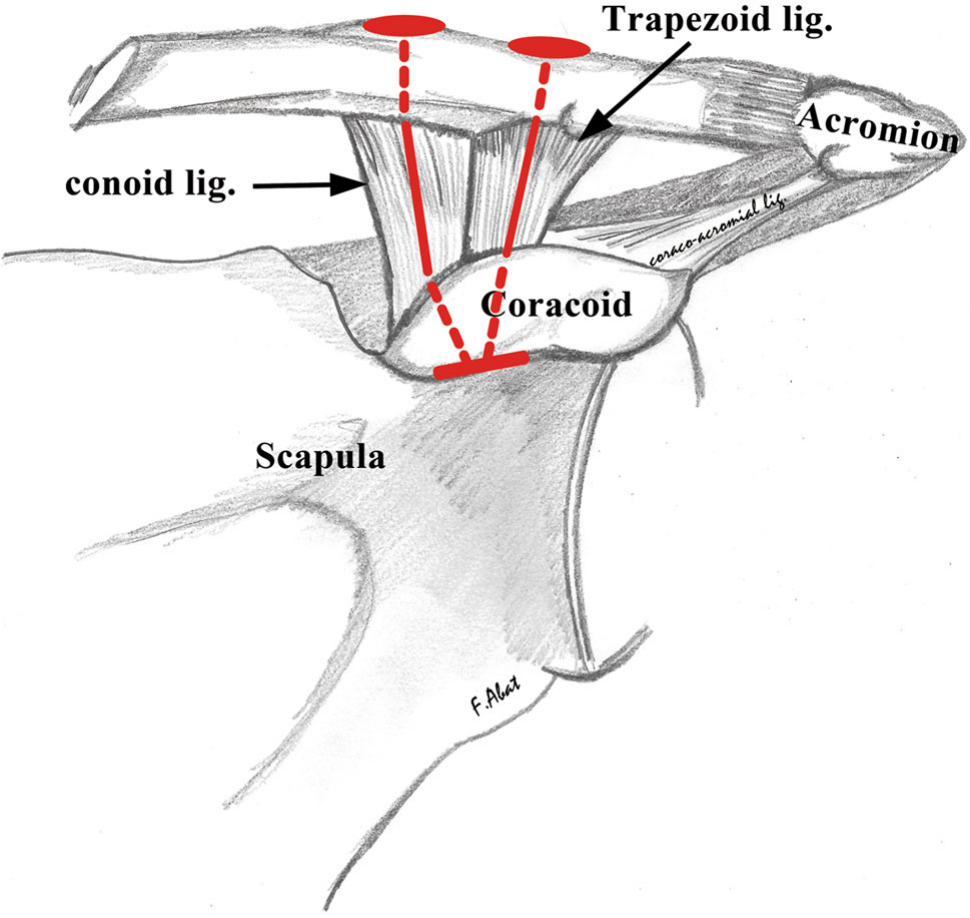
Scheme for anatomic repair of the coracoclavicular ligaments in a “V” configuration. Note the arrangement of a single CC suspension device with two tunnels in the clavicle and one in the coracoid

In the reconstruction with a single tunnel in the coracoid (group III) for isometric repair of the CC ligaments in a “V” configuration (Fig. 2), two tunnels were created in the clavicle at the usual insertion of the conoid and trapezoid ligaments and one was made at the base of the coracoid (at the midpoint of the insertion of both ligaments). The CC suspension device was put in place with the titanium component locked into the base of the coracoid, passing through the tunnel inversely. Each loop of the device was then passed through the corresponding tunnel in the clavicle so as to obtain a repair of the CC ligaments with a single implant in a “V” configuration.

### Biomechanical study protocol

The studied cadaveric pieces were placed in a universal testing machine (Electro Puls 3000, Instron, Boulder, MA, USA) with the clamping jaws vertical. The base of the scapula was fixed to the clamp by a compression system using two plates with screw tips to ensure proper fixation. By means of brackets, a bar contacting the upper edge of the scapula was fitted to prevent vertical movement.

To analyze the vertical behavior of the CC suspension systems, two rings were placed in the clavicle (one outside and one inside the fixations), which were connected by two chains to the vertical movement clamp. The chains allowed the traction system to be placed at the same distance. The traction test was performed at a speed of 15 mm/min. Pre-tensioning was performed at 15 N before the displacement of the bar of the testing machine was initiated. The test was stopped when the tensile force dropped by 60 % of the maximum applied force (Fmax 60 %) or when mobility of the part or implant failure was observed. In each test, the maximum breaking force (in N) was obtained. Group I (control) was tested first, thereby obtaining the reference values for a healthy shoulder. Groups II and III were tested later.

### Statistical analysis

Mean values were calculated along with their standard deviations. Comparison was performed with an analysis of variance using the Tukey post-hoc test. SPSS (v.21.0) software was used. The level of significance was the usual 5 % (*α* = 0.05, bilateral).

## Results

The results obtained with the vertical traction biomechanical test to evaluate the maximum breaking force are shown in Table 1.

**Table 1:** Maximum breaking force in the vertical traction biomechanical test for each cadaveric piece

	Group I Control	Group II	Group III
1	534.44	412.43	374.12
2	529.94	351.99	274.10
3	245.85	278.75	533.11
4	253.49	939.37	371.50
5	464.81	*	300.05
6	635.59	*	210.30

Values expressed in Newtons (N)

In group I and the control group (*n* = 6), the CC ligaments tore in all specimens upon reaching a maximum of 635.59 N and a minimum of 245.85 N. In group II (two tunnels and two fixations), two of the pieces were torn by the scapular fixation, so they were discarded. In the remaining four, the maximum force achieved was 939.37 N and the minimum was 278.75 N. In group III (two fixations and a single coracoid tunnel), the maximum value was 533.11 N and the minimum value was 210.30 N.

Upon performing a cluster analysis (Table 2), group I showed an average peak force of 444.0 N (SD 160.16) and group II averaged 495.6 N (SD 300.83). Group III had an average of 343.9 N (SD 111.46). A comparison of the three groups did not show any significant differences (ANOVA, *p* = 0.446), although the clear decline in resistance in group III is worth noting. Furthermore, no subsequent peer comparison indicated a significant difference from the overall value (Tukey post-hoc test, group I vs II, *p* = 0.906, group I vs III, *p* = 0.638, and group II vs III, *p* = 0.448).

**Table 2:** Mean values of maximum force according to study groups

Group	Average	SD	CV (%)	*n*
I	444.0	160.16	36.1	6
II	495.6	300.83	60.7	4
III	343.9	111.46	32.4	6
Total	419.4	186.73	44.5	16

Values expressed in Newtons (N)

## Discussion

The main finding of this study was that the anatomic repair of the CC ligament with a double system (double tunnel in both the clavicle and coracoid) is biomechanically more like the AC joint than the AC repair with a single CC system in a “V” configuration is. Moreover, AC repair with a single CC system in a “V” configuration does not appear to be biomechanically sufficient, as it shows a clear tendency to offer less resistance. These findings confirm the hypothesis of the present study. We believe that compliance with these anatomical and biomechanical objectives allows for fewer recurrent subluxations and less residual pain, leading to a better clinical outcome.

A large number of AC dislocation repair techniques have been described, but there is still controversy over what the standard technique should be. Generally, repair focuses on reinforcing the CC ligaments with non-absorbable sutures, screws, pins, plates, or other methods of internal fixation [13–15]. Repairs with tendon grafts or fixation devices are based on the Weaver–Dunn technique and its variations [16, 17]. Initially, these coracoclavicular suspension devices were described for tibiofibular syndesmosis repair, but they have since been used in AC joint reconstruction too [18, 19]. Authors such as Salzmann [5] and Walz [12] argue that the placement of two CC suspension systems as replacements for the conoid and trapezoid ligaments is required to achieve proper primary stability. The precise anatomy of these two CC ligaments has already been described: the length of each ligament should be about 10 mm, giving a distance of 10–15 mm between the clavicle and coracoid [1, 5, 9, 12]. In agreement with a study by Breslow [20], the AC capsule and its ligaments work together to maintain horizontal stability, while the CC ligaments limit vertical displacement. Dimakopoulos et al. [21] were the first to provide clinical data on double-bundle repair for acute AC dislocations. The Mazzoca group [3, 7] described the open clamp technique with a semitendinosus tendon which, despite using an anatomic collarbone implementation, only uses a single point of traction on the coracoid. Lafosse et al. [22] reported a modified Weaver–Dunn technique performed arthroscopically, while Choi et al. [23] described procedures that use suture fixations to repair acute dislocations of the AC complex, with two sutures placed in the anatomic position to provide primary stability of the AC and CC ligaments. Recently, Tomlinson [24] and Baumgarten [25] described the anatomic repair of the CC complex using tendon grafts in the form of a cerclage around the coracoid with anatomic fixation under the clavicle. Rehbein et al. [26] reported a transosseous suture technique with a cerclage in the AC and CC in an anatomic position.

Morrison et al. [15] suggest that a simple loop around the coracoid to repair the CC ligaments can cause the final position of the left clavicle to be displaced anteriorly. In the present study, we proposed that the best reconstruction is performed in the anatomical arrangement emulating the conoid and trapezoid ligaments. To achieve this, we tested two configurations, one of which was a “V” that emulated the fixation in the collarbone but with one tunnel placed at the isometric point of the coracoid, and the other a configuration with a double tunnel in the clavicle and a double tunnel in the coracoid that used two coracoclavicular suspension devices.

Chernchujit et al. [4] reported CC ligament tension results of 578 N, whereas they reached a value of 767 N with a double FiberWire^®^ suture. Furthermore, Walz et al. [12] achieved a tension of 982 N using two coracoclavicular suspension systems (TightRope^®^). Wellmann et al. [27] reported a value of 663 N for a repair performed with polydioxanone (PDS), similar to the value recently reported by Martetschläger et al. [28]. Our study showed mean values for intact ligaments of 444.0 N (maximum 635.59 N), while the mean value for anatomic repairs involving double tunnels in the coracoid was 495.51 N (maximum 939.37 N). Although our study yielded lower values in terms of the average tension obtained with the repair compared to other reference works such as Motamedi et al. [29] and Wellmann et al. [27], the results reported here are very similar to those obtained in specimens with an intact acromioclavicular joint.

The main limitation of this study is that it is a cadaveric biomechanical study, so it inherently differed from the normal clinical situation. Nonetheless, this is a rigorous, well-controlled, and reproducible work. Another weakness is that we did not test the failure of the repair in combined craniocaudal, anterior–posterior, and rotational traction. The average age (58 years) of the donors of the cadaveric parts used is, however, comparable to those presented in previous studies, and is substantially higher than the normal average age at presentation of acromioclavicular dislocations (20 years). Since it has been shown that the mechanical qualities of the ligaments and bones deteriorate over the years, better results would be expected in younger individuals. One other weakness is that two specimens were lost from group II.

The results obtained in this study indicate that repair with a synthetic double CC suspension device with double tunneling in the coracoid as well as the clavicle gives vertical traction biomechanical results that resemble those of the native AC joint.
